# Genetic variants in the transcription regulatory region of *MEGF10* are associated with autism in Chinese Han population

**DOI:** 10.1038/s41598-017-02348-1

**Published:** 2017-05-23

**Authors:** Zhiliu Wu, Jian Qin, Yang You, Yuanlin Ma, Meixiang Jia, Linyan Wang, Tianlan Lu, Weihua Yue, Yanyan Ruan, Dai Zhang, Jun Li, Lifang Wang

**Affiliations:** 1 Institute of Mental Health, The Sixth Hospital, Peking University, Beijing, P. R. China; 2 Key Laboratory of Mental Health, Ministry of Health & National Clinical Research Center for Mental Disorders (Peking University), Beijing, P.R. China; 3 Central Laboratory, Renmin Hospital, Wuhan University, Wuhan, Hubei P. R. China; 4 Peking-Tsinghua Center for Life Sciences, Peking University, Beijing, P. R. China; 5 PKU-IDG/McGovern Institute for Brain Research, Peking University, Beijing, P. R. China

## Abstract

Multiple epidermal growth factor-like-domains 10 (*MEGF10*), a critical member of the apoptotic engulfment pathway, mediates axon pruning and synapse elimination during brain development. Previous studies indicated that synaptic pruning deficit was associated with autism-related phenotypes. However, the relationship between *MEGF10* and autism remains poorly understood. Disease-associated variants are significantly enriched in the transcription regulatory regions. These include the transcription start site (TSS) and its cis-regulatory elements. To investigate the role of *MEGF10* variants with putative transcription regulatory function in the etiology of autism, we performed a family-based association study in 410 Chinese Han trios. Our results indicate that three single nucleotide polymorphisms (SNPs), rs4836316, rs2194079 and rs4836317 near the TSS are significantly associated with autism following Bonferroni correction (*p* = 0.0011, *p* = 0.0088, and *p* = 0.0023, respectively). Haplotype T-A-G (rs4836316-rs2194079-rs4836317) was preferentially transmitted from parents to affected offspring (*p*_permutation_ = 0.0055). Consistently, functional exploration further verified that the risk allele and haplotype might influence its binding with transcription factors, resulting in decreased transcriptional activity of *MEGF10*. Our findings indicated that the risk alleles and haplotype near the *MEGF10* TSS might modulate transcriptional activity and increase the susceptibility to autism.

## Introduction

Autism is an early-onset neurodevelopmental disorder that is characterized by impaired social interaction and communication, repetitive behavior, and restricted interests^1^. The core symptoms of autism usually occur before three years of age. Epidemiological studies indicate that genetic factors strongly increase the risk of autism^2, 3^. Additionally, twin studies indicate that the estimated heritability of autism may be as much as 90%^4^. However, the pathogenesis of autism remains largely unclear.

Abnormal numbers and connectivity of synapses caused by synaptic pruning deficit might play a marked role in the pathogenesis of autism^5, 6^. Previous functional studies have revealed that deficient synaptic pruning is associated with autism-related phenotypes, such as deficits in social interaction and increased repetitive-behavior^5, 7^. In addition, previous genetic studies have reported that individuals affected with autism carry mutations or structural variants in several genes associated with synaptic formation and function, including *NRXN1* (Neurexin1), *NLGN3* (Neuroligin3), *SHANK2* (SH3 and multiple ankyrin repeat domains 2), and *SHANK3* (SH3 and multiple ankyrin repeat domains 3)^8–13^. These genes regulate neuronal activity by modulating synaptic number or strength^14–18^. Mice carrying mutations of these genes presented not only abnormal synaptic function or morphology but also abnormal social interaction^19, 20^. These lines of evidence suggest that synaptic dysregulation might contribute to the etiology of autism.

Previous studies have indicated that defects in synaptic pruning might cause altered synaptic density and/or shape of the autistic brain^5, 21^. Multiple epidermal growth factor-like-domains 10 (*MEGF10*), which is a critical member of the apoptotic engulfment pathway, modulates synaptic number and function via synaptic pruning and phagocytosis in the postnatal brain. A mouse model has suggested that *MEGF10* might play a critical role in synapse elimination via astrocytes in the postnatal brain. Mice deficient in the Megf10 pathway displayed dysfunctional and weak synapses and failed to retain excess functional synapse during the postnatal period^22^. A recent study in mice also found that dysfunction of *Megf10* impaired phagocytosis mediated by astrocytes in the developing cerebellum^23^. Moreover, previous studies have indicated that *cell death abnormal 1* (*CED-1*) and *draper* (*DRPR*), which represent mammalian orthologues of *MEGF10* in *Caenorhabditis elegans* and *Drosophilia melanogaster* respectively, might regulate the clearance of apoptotic cells, axon pruning and neuronal degeneration^24–26^. Axon pruning and neuronal degeneration mediated by *CED-1* modulate the number of synapses and synaptic connectivity^27, 28^. Therefore, a critical role in the regulation of synaptic number and function suggests that *MEGF10* is a candidate gene ﻿for autism.

*MEGF10* is located on chromosome 5q33. Linkage analysis revealed that 5q33.1 was associated with motor/speech phenotype in autism^29^. An association study suggested that *paired like homeodomain 1* (*PITX1*), which is located adjacent to *MEGF10*, might be an autism susceptibility gene^30^. Furthermore, other association studies have implicated that *MEGF10* and other genes in the apoptotic engulfment pathway are significantly associated with schizophrenia^31, 32^. Autism and schizophrenia share several clinical features including impaired executive function and deficits in social function^33, 34^. Several lines of evidence indicate that schizophrenia and autism share similar candidate genes, at least partially, including *CACNA1C* ^35, 36^, *DISC1* ^37, 38^, *SHANK3* ^9, 39^, *NRXN1* ^40, 41^, and *NTNG1* ^42, 43^, which are related to the modulation of the synapses. However, the relationship between *MEGF10* and etiology of autism remains poorly understood.

Recently, a systematic study revealed that disease-associated SNPs were significantly enriched in the functional regions annotated by the ENCODE consortium and Roadmap project^44, 45^. Several functional regions, including the transcription start site (TSS) and its cis-regulatory elements, play a critical role in modulating gene transcription. Moreover, SNPs in this region often represent significant expression Quantitative Trait Loci (eQTL) signals that are related to mRNA abundance^46^. On collectively considering both the role of *MEGF10* and its regulation of synaptic pruning and the relationship of regulatory functional variants with disease, we hypothesized that variants in *MEGF10* with putative transcriptional regulatory function might play a role in the pathogenesis of autism. To elucidate the genetic relationship between *MEGF10* and autism, we primarily selected the three SNPs in the TSS and an additional two SNPs in the non-regulatory region. Then, we performed a family-based association study involving 410 Chinese Han family trios to investigate the association of five selected SNPs with autism. Furthermore, several functional assays were used to verify the regulatory function of three variants in the TSS of *MEGF10*.

## Results

### Quality control

According to the SNP selection criteria described in the Methods section of this article, seven SNPs (i.e., rs2408868, rs10519929, rs3812058, rs10519930, rs1363387, rs4836316 and rs2194079) were located in the active transcription start site and cis-regulatory elements. Of these SNPs, rs10519929, rs3812058 and rs10519930 that bind to TF were not selected due to their MAF having a value less than 0.05 in the CHB population of the Hapmap project (MAF = 0.018, 0.045 and 0.018 respectively). Rs4836316 and rs2194079 were completely linkage disequilibrium with rs1363387 and rs2408868 respectively. The number of binding TF motifs change for rs4836316 was more than that for rs1363387 (Supplementary Table S1). Moreover, the change of TF motif log-odds (LOD) score of rs2194079 was greater than that of rs2408868. Thus, rs4836316 and rs2194079 were preferentially selected as tag SNPs. Meanwhile, we selected rs4836317 to construct haplotype with rs4836316 and rs2194079. An additional two SNPs (i.e., rs9327438 and rs6866678) in the non-regulatory region were selected to compare their association with three selected SNPs near the TSS region. These selected SNPs covered 60.5% of the whole gene. The mean distance between each of the neighboring SNPs was 34.2 Kb.

A total of 41 missing genotypes consisted of three genotypes for SNP rs4836316; four for SNP rs2194079; 13 for SNP rs4836317; 16 for SNP rs9327438 and five for SNP rs6866678. The call rate of each genotype in the study subjects is greater than 95% which is for Sanger sequencing and the Agena Bioscience platform. SNPs frequencies are displayed in Table 1. None of the genotypic distributions of these SNPs in unaffected parents and affected offspring deviated from HWE (Supplementary Table S3). The genotyping concordance rate for the Agena Bioscience platform and Sanger sequencing in 10% of the samples exceeded 99%.

**Table 1:** Association analyses of 5 SNPs in *MEGF10* in 410 trios using FBAT in an additive model.

Marker	Chromosome	Allele	T : U	Allele frequency	Fam	S-E (S)	Var (S)	Z	*P*
rs4836316	5:127292183	T	199 : 140	0.707	269	30.00	85.00	3.254	**0**.**0011**
		G	140 : 199	0.293	269	−30.00	85.00	−3.254	**0**.**0011**
rs2194079	5:127292406	A	230 : 177	0.585	309	26.50	102.25	2.621	**0**.**0088**
		G	177 : 230	0.415	309	−26.50	102.25	−2.621	**0**.**0088**
rs4836317	5:127293079	G	196 : 140	0.707	266	28.00	84.00	3.055	**0**.**0023**
		T	140 : 196	0.293	266	−28.00	84.00	−3.055	**0**.**0023**
rs9327438	5:127387870	G	216 : 173	0.609	300	21.50	97.25	2.180	0.029
		A	173 : 216	0.391	300	−21.50	97.25	−2.180	0.029
rs6866678	5:127432269	A	230 : 189	0.547	309	20.50	104.75	2.003	0.045
		G	189 : 230	0.453	309	−20.50	104.75	−2.003	0.045

Abbreviations: T, transmitted; U, untransmitted; Fam, number of informative families; S, test statistics for the observed number of transmitted alleles; E(S), expected value of S under the null hypothesis (i.e., no linkage and no association).

Note: T : U is the ratio of transmissions to non transmissions of the allele; *P* value in bold font indicates persistent statistical significance after Bonferroni correction.

### Putative regulatory function and eQTL of selected SNPs

The rVarbase predicted that rs4836316, rs2194079 and rs4836317 were located in the active TSS region which might overlap with several cis-regulatory elements. In addition, HaploReg indicated that they disrupt binding motifs of several transcriptional regulatory factors (Supplementary Table S1). The eQTL analysis from the BRAINEAC database showed that all selected SNPs were associated with the expression level of *MEGF10* in several brain regions (Supplementary Fig. S3). Remarkably, rs4836316 and rs4836317 were significantly associated with the expression of *MEGF10* in the hippocampus after the *p* value was corrected by a false discovery rate (FDR) of <1% (*p* = 1.1e-6, *p* = 1.2e-7, respectively) (Supplementary Fig. S3). In the GTEx database, the multi-tissue (53 human tissues including 10 primary brain regions) eQTL comparison for five selected SNPs revealed that several brain regions, such as the cerebellum and frontal cortex, were predicted to show a strong eQTL effect (post-prob >0.9) (Supplementary Table S5).

### SNP association and haplotype association analyses

Under an additive model, univariate (single marker) tests showed that the T allele of rs4836316, the A allele of rs2194079 and the G allele of rs4836317 were significantly preferentially transmitted from parents to the affected offspring (rs4836316: T > G, Z = 3.254, *p* = 0.0011; rs2194079: A > G, Z = 2.621, *p* = 0.0088; rs4836317: G > T, Z = 3.055, *p* = 0.0023) (Table 1). Moreover, the T allele of rs4836316 and the G allele of rs4836317 were preferentially transmitted from parents to offspring under a dominant model (rs4836316: T > G, Z = 2.762, *p* = 0.0057; rs4836317: G > T, Z = 2.752, *p* = 0.0059) (Supplementary Table S4). The statistical significance persisted even after Bonferroni correction (*p* = α/n = 0.05/5 = 0.01).

**Figure 1:**
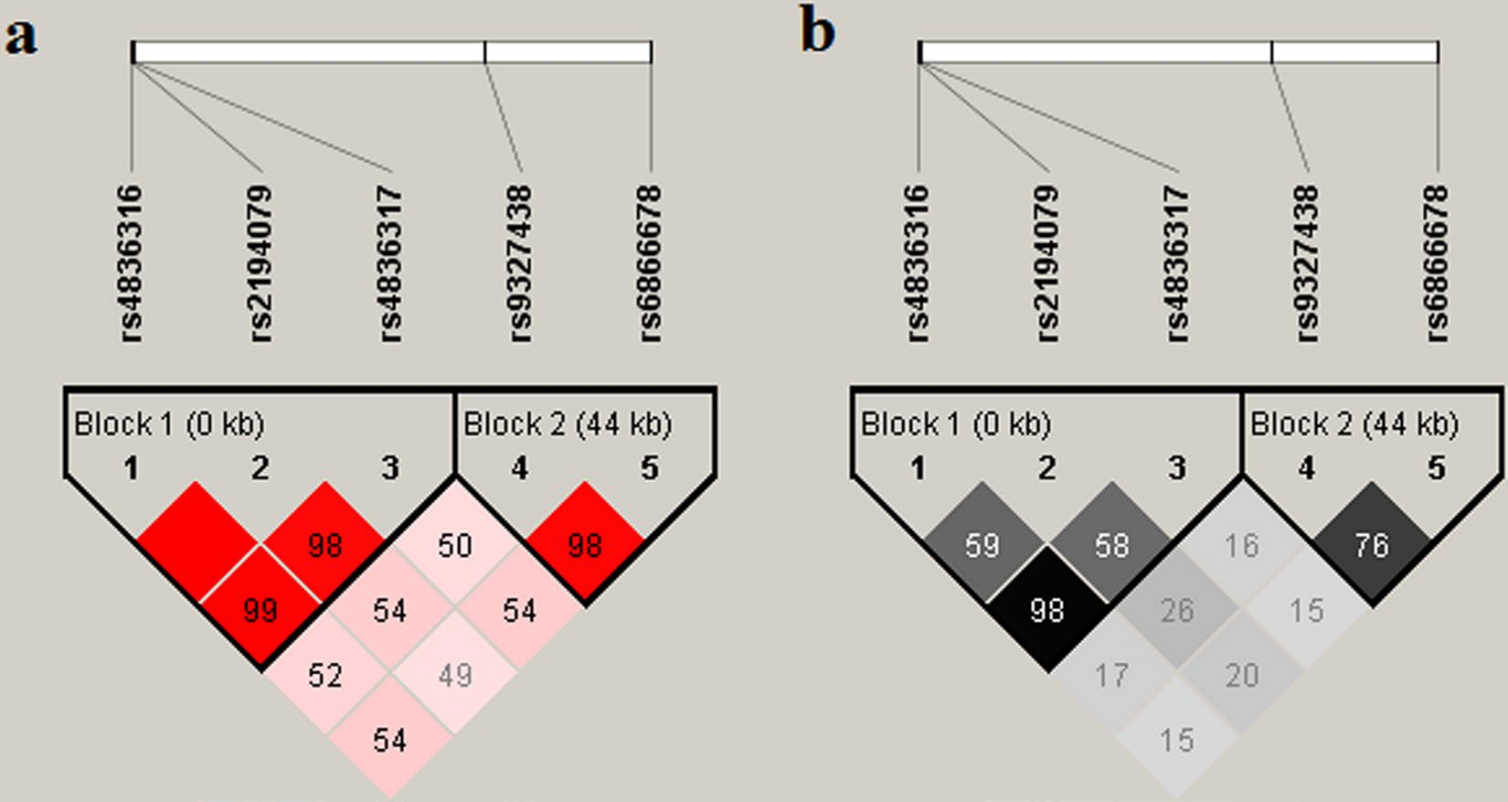
Linkage disequilibrium (LD) block constructed from 5 SNPs in *MEGF10*. (**a**) Markers with linkage disequilibrium (LD) (*D*’ < 1 and LOD >2) are shown in red through pink (color intensity decreases with decreasing *D*’ value). *D*’ value shown in each square represents pairwise LD relationship between the two polymorphisms. *D* prime values of 1.0 are never shown (the box is empty). The LD plot was generated using the Haploview program. (**b**) The value of *r* ^*2*^ is shown in each square. Black square indicated *r* ^*2*^ = 1 (i.e. perfect LD between a pair of SNPs). Markers with LD are shown in black to grey (color intensity decreases with decreasing *r* ^*2*^ value).

As shown in Fig. 1b, the r-square value indicated that five selected SNPs constructed two potential LD blocks. In addition, rs4836316, rs2194079 and rs4836317 were located in block 1, while others (i.e., rs9327438 and rs6866678) were located in block 2. Specific and global haplotype association tests were carried out only for the LD blocks. All haplotypes were constructed using the sliding window approach. We found that haplotype T-A-G (rs4836316-rs2194079-rs4836317; Z = 2.779, *p* = 0.0055, Global *p* = 0.0079) (Table 2) was transmitted more than haplotype G-G-T (Z = −3.087, *p* = 0.0020, Global *p* = 0.0079) (Table 2). These results remained significant after permutation testing (n = 10,000, *p* = 0.003). In addition, the haplotypes T-A (rs4836316-rs2194079) and the haplotype A-G (rs2194079-rs4836317) that was constructed from risk alleles also displayed preferential transmission from parents to affected offspring (Z = 2.595, *p* = 0.0094, Global *p* = 0.0058; Z = 2.780, *p* = 0.0054, Global *p* = 0.0020, respectively) (Table 2). However, the haplotype G-A that was composed of rs9327438 and rs6866678 did not show significant preferential transmission (Z = 1.907, *p* = 0.056, Global *p* = 0.014) (Table 2).

**Table 2:** Results of haplotype analyses of 5 SNPs in *MEGF10* in an additive model.

Marker	Haplotypes	T : U	Freq	Fam	S-E (S)	Var (S)	Z	*P*	Global *P*	Permutation^1^ *P*
rs4836316-rs2194079	T-A	231.5 : 178.5	0.575	266	26.50	104.25	2.595	**0**.**0094**	**0**.**0058**	**0**.**0033**
	G-G	141.0 : 200.0	0.306	237	−30.00	87.50	−3.207	**0**.**0013**		
	T-G	86.5 : 80.5	0.119	146	3.50	41.75	0.542	0.59		
rs2194079-rs4836317	A-G	231.6 : 178.3	0.573	274	28.00	101.49	2.780	**0**.**0054**	**0**.**0083**	**0**.**0034**
	G-T	142.2 : 197.9	0.302	236	−28.50	84.72	−3.096	**0**.**0020**		
	G-G	87.7 : 83.4	0.121	145	2.00	40.95	0.312	0.75		
	A-T	n/a	0.004	29	−1.50	1.25	−1.344	0.18		
rs4836316-rs2194079-rs4836317	T-A-G	231.7 : 178.3	0.574	257	28.00	101.50	2.779	**0**.**0055**	**0**.**0079**	**0**.**0035**
	G-G-T	142.0 : 198.0	0.302	228	−28.50	85.25	−3.087	**0**.**0020**		
	T-G-G	86.5 : 80.8	0.119	141	2.00	40.50	0.314	0.75		
	T-A-T	n/a	0.004	5	n/a	n/a	n/a	n/a		
	G-G-G	n/a	0.001	3	n/a	n/a	n/a	n/a		
rs9327438-rs6866678	G-A	229.1 : 194.9	0.536	272	20.00	109.99	1.907	0.056	0.014	0.012
	A-G	179.3 : 221.7	0.397	270	−24.50	102.23	−2.423	0.015		
	G-G	49.0 : 45.8	0.064	92	2.00	22.97	0.416	0.68		
	A-A	n/a	0.004	28	2.50	1.25	2.235	0.025		

Abbreviations: T, transmitted; U, untransmitted; Freq, Estimation of haplotype frequencies; Fam, number of informative families; S, test statistics for the observed number of transmitted alleles; E(S), expected value of S under the null hypothesis (i.e., no linkage and no association); n/a: not applicable.

Note: ^1^The number of permutation is 10,000; whole marker permutation test using chisq sum *p* value; T : U is the ratio of transmissions to non transmissions of the allele; *P* value in bold font represents statistical significance after permutation test.

### Regulatory effects of the haplotype constructed from three risk SNPs on the transcriptional activity

**Figure 2:**
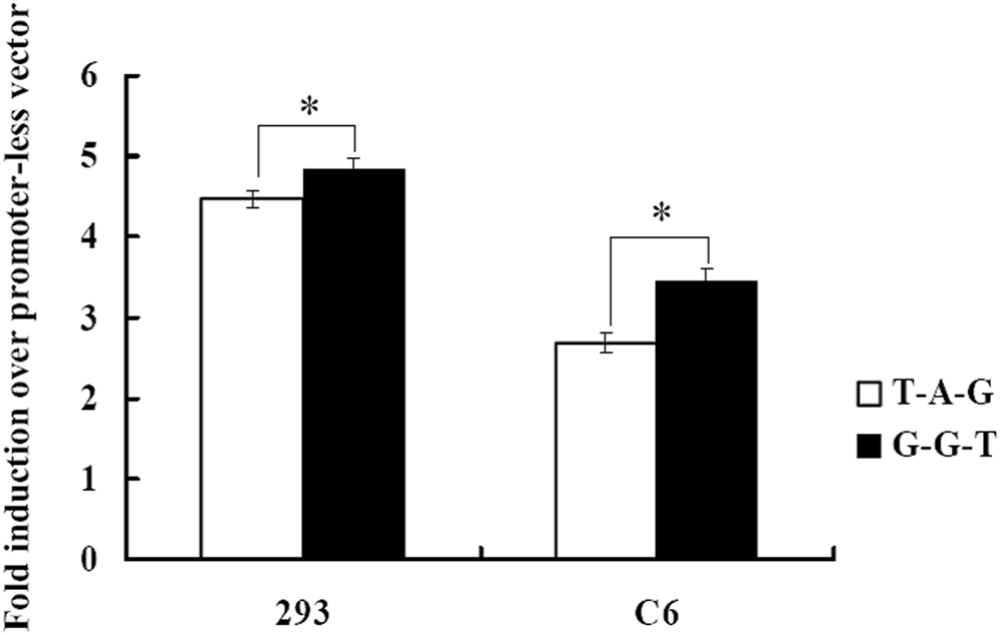
Regulatory effects of the haplotypes constructed from rs4836316, rs2194079 and rs4836317 on the transcriptional activity. The transcriptional activity was decreased when the risk haplotype T-A-G in 293 and C6 cell lines. Bars are presented as fold-induction compared with promoter less vector. Error bars represent standard error of mean (SEM) (*n* = 5). *P < 0.05.

The 1.5 kb fragments that contained haplotype T-A-G (rs4836316-rs2194079-rs4836317) and G-G-T were independently cloned upstream of the luciferase reporter gene (pGL3-Basic). They were transfected into both 293T and C6 cells. The reporter construct containing the haplotype T-A-G showed decreased luciferase activity as compared with the reporter construct that contained the haplotype G-G-T in both cell lines (*P* < 0.05 for both cell lines) (Fig. 2). These results indicated that the haplotype T-A-G was less efficient in driving transcription than the haplotype G-G-T. Results of functional prediction were partially validated by this experiment.

### Protein binding ability of three risk SNPs

**Figure 3:**
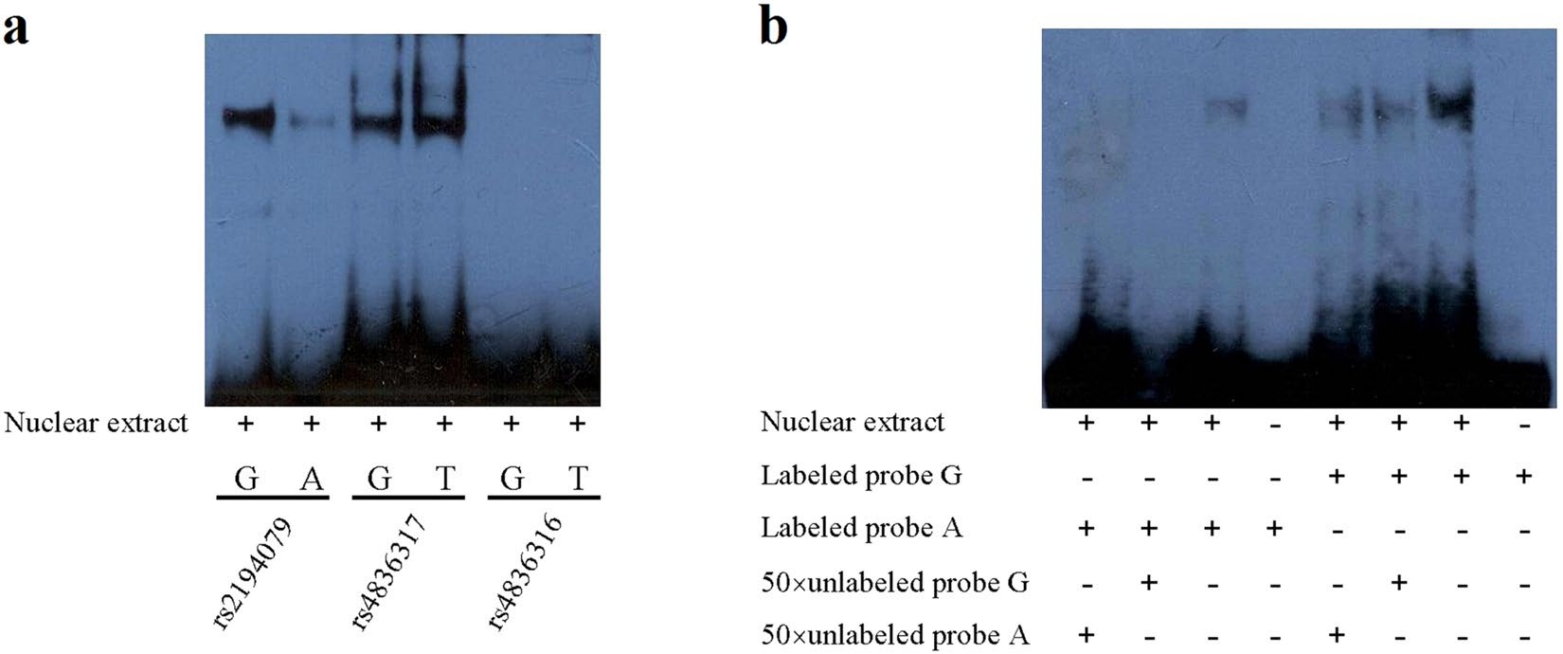
Results of electrophoretic mobility shift assays for rs4836316, rs2194079 and rs4836317. Electrophoretic mobility shift assays were performed with biotinylated oligonucleotides and nuclear extracts isolated from C6 cell line to investigate the binding of associated SNP with nuclear extract. (**a**) The labeled probes containing variants of rs2194079, rs4836316, rs4836317 were incubated with the nuclear proteins. SNP rs4836316 showed no band shift. Both rs2194079 and rs4836317 showed band shifts. The allele G of rs2194079 showed preferential binding with the nuclear extract. (**b**) Specificity was determined by competition with 50-fold unlabeled probes. Protein-DNA complexes were observed for both alleles. However, preferential binding of G allele in rs2194079 was observed. The “+” and “−” indicate the presence and absence of nuclear extract or probe.

As shown in Fig. 3, band retardation was observed in the presence of nuclear proteins in lanes that were represented by rs2194079 and rs4836317. The binding activity of the A allele in rs2194079 was weaker than the G allele, while the alleles of rs4836317 were almost identical. The EMSA results showed that the A allele of rs2194079 is relatively weak in binding nuclear proteins as compared with the G allele of rs2194079.

### Expression pattern of *MEGF10* in human brain and peripheral blood of autistic individuals

The expression of *MEGF10* in 13 human brain regions was higher than that found in other human organs or tissues, and especially in the spinal cord, basal ganglia, substantia nigra and the hippocampus (Supplementary Figs S2 and S4). As shown in the figure from HBT database, during developmental stages, *MEGF10* expression in the hippocampus and other brain regions was initially decreased and then progressively increased. After birth, its expression was stable and it was sustained at a relatively high level in different regions of the brain throughout life (Supplementary Fig. S5a). The dynamic expression of *MEGF10* in 11 areas of the neocortex was similar to the expression that found in other brain regions (Supplementary Fig. S5b). In the GEO dataset from Alter *et al*.^47^, the normalized expression value of *MEGF10* in autistic individuals was lower than that found in healthy controls (*p* < 0.05) (Supplementary Fig. S6).

## Discussion

We studied the genetic association of five SNPs in *MEGF10* with autism in a Chinese Han cohort. The results indicated that three SNPs (i.e., rs4836316, rs2194079 and rs4836317) in the transcription start site were significantly associated with autism. Our observations also indicated that haplotype T-A-G that was constructed from these SNPs might be a risk factor for autism.

A previous genome-wide association study (GWAS) of the Psychiatric Genomic Consortium (PGC) that included 4788 trio cases and 4788 trio pseudocontrols demonstrated that thirty SNPs in *MEGF10* were nominally associated with autism in a CEU (Northern and Western European Ancestry in Utah) population^35^ (Supplementary Table S7). Of these SNPs, rs10519929, rs10519930 and rs17165105 were located in the same haplotype block which contained three positive SNPs (i.e., rs4836316, rs2194079 and rs4836317) that we tested (Supplementary Fig. S1). All six SNPs were mapped to haplotype spanning the transcription start site and were predicted to possess similar regulatory function. The rVarbase and HaploReg database also indicated that rare variants such as rs10519929 and rs10519930 which were located approximately 2Kb upstream of the risk haplotype T-A-G (rs4836316-rs2194079-rs4836317) might also disrupt transcriptional factor binding sites (Supplementary Table S1 and Supplementary Fig. S7). This evidence suggests that disrupted transcriptional regulation of *MEGF10* contributes to autism risk. In our study, functional experiments verified that the risk alleles and haplotype might affect binding with transcription factors, which would result in decreased transcriptional activity of *MEGF10*. The HaploReg database indicated that the risk haplotype could lead to reduced transcription factor binding consistent with our results. Based on the putative regulatory function of these common and rare variants in the TSS proximal region of *MEGF10*, these variants would seem to increase autism risk through reducing *MEGF10* dosage. A previous transcriptome study showed that *MEGF10* is strongly expressed in glial cells, especially astrocytes^48^. Compared to haplotype T-A-G (rs4836316-rs2194079-rs4836317), haplotype G-G-T increased the reporter expression in the C6 cell-line, derived from rat glial cells. Meanwhile, the specific binding of nuclear factors to rs2194079 supported the differential function of each haplotype. This evidence suggests that the risk haplotype T-A-G might decrease the transcription of *MEGF10* by disrupting binding with transcription factors found in human glial cells. Moreover, these three SNPs represented positive eQTL signals in primary brain regions and decreased expression of *MEGF10* was observed in the peripheral blood of autistic individuals from a GEO profile dataset.

During the postnatal period, the low expression of *MEGF10* in the brain might affect the modulation of synaptic number or function. Previous functional experiments conducted in *Megf10* ^−/−^ mice indicated a critical role in synapse elimination in the developing and adult brain. In developing mice, *Megf10-*deficient astrocytes led to a failure in retaining excess functional synapses. During adulthood, astrocytes enriched in *Megf*10 continuously phagocytosed both excitatory and inhibitory synapses^22^. *MEGF10* is evolutionarily conserved and is a key member of phagocytic pathways that contributes to axon pruning by glial cells in *Drosophilia melanogaster* ^25^. Further, functional investigation has indicated that synaptic pruning deficits caused by aberrant autophagy have contributed to the development of ASD-like social behavior^5^. Therefore, the dampened *MEGF10* expression may trigger synaptic dysregulation and disrupt the rate of synapse formation and pruning, thus playing a role in the pathogenesis of autism.

In this study, we established a genetic relationship between *MEGF10* and autism in the Chinese Han population. Further, for the risk haplotype T-A-G spanning the TSS, we explored its function in regulating the transcriptional activity of *MEGF10*. Notably, *MEGF10* is intolerant of heterozygous loss of function (LoF) based on the probability of LoF intolerance (pLI) score of 0.99 from the ExAC database. This indicates that the inherited contribution to autism etiology of this gene would have to be explored through putatively hypomorphic variants only. Sequencing rare mutations of *MEGF10* in autistic individuals is also needed in future studies. In individuals carrying the risk alleles of rs4836316, rs2194079 and rs4836317, the low level of *MEGF10* expression should be further validated in the developing brain. Furthermore, functional studies of *MEGF10* in mice are required to illustrate its relationship with autism.

In summary, our findings suggest that *MEGF10*, which mediates axon pruning and synapse elimination during brain development, is associated with autism in the Chinese Han population. In addition, the haplotype T-A-G composed of rs4836316, rs2194079 and rs4836317 that near the TSS seems to affect autism risk by modulating the transcriptional activity of *MEGF10*.

## Methods

### Ethics statement

All subjects (including singleton autistic disorder children and their unaffected biological parents) provided written informed consent to participate in this study. This study was approved by the Ethics Committee of the Institute of Mental Health, The Sixth Hospital, Peking University (P. R. China). All methods were performed in accordance with approved guidelines.

### Subjects

All subjects were recruited at the Institute of Mental Health, The Sixth Hospital of Peking University, of the P. R. China. They were of Chinese Han descent for at least three generations (i.e. four Chinese Han grandparents). All patients fulfilled the criteria of the Diagnostic and Statistical Manual of Mental Disorders, Fourth Edition (DSM-IV) for autistic disorders. The diagnosis of autism was established by two senior psychiatrists. The clinical features of patients were assessed using the Childhood Autism Rating Scale (CARS)^49^ and Autism Behavior Checklist (ABC)^50^. Children with CARS scores ≥35 and ABC score ≥53 were included in this study.

Children with fragile X syndrome, Asperger’s syndrome, Rett syndrome, tuberous sclerosis, or a previously identified chromosomal abnormality, dysmorphic features, or any other neurological condition were excluded from the study. Individuals with other severe psychiatric disorders or familial inherited diseases were also excluded from this study. Finally, 410 nuclear trios were included. Of the 410 probands affected by autism, 383 were male and 27 were female. The median age of the children at diagnosis was 5.5 years (range 3–17 years).

### SNP selection

Information that was related to single nucleotide polymorphisms (SNPs) of *MEGF10* in CHB (Chinese Han, Beijing) was downloaded from the dbSNP (http://www.ncbi.nlm.nih.gov/SNP/) and the international HapMap project in phase II and III (ftp://ftp.ncbi.nlm.nih.gov/hapmap). Minor allele frequency (MAF) and *p*-value derived from the Hardy-Weinberg Equilibrium (HWE) of all SNPs in the CHB population exceeded 5%, respectively. Tagger module in Haploview version 4.2 was used to optimize SNP selection.

Functional annotations and characteristics for all SNPs located in the haplotypes which included the TSS of *MEGF10* were displayed in Supplementary Table S1. As shown in Supplementary Fig. S1, 18 SNPs were located in this haplotype block. Utilizing four online databases, there are three basic criteria that were required to select SNPs having potential transcriptional regulatory function: 1. based on the results from the rVarbase databases (http://rv.psych.ac.cn/)^51^, SNPs that were located proximal to the TSS and in annotated cis-regulatory elements were preferred to have regulatory function. The active TSS annotated by rVarbase spans from 908 bp upstream of *MEGF10* TSS to 3305 bp downstream of that. SNPs in the annotated cis-regulatory elements, including TF-binding region and chromosome interactive region of this span, were preferentially selected. 2. For the SNPs that located in both active TSS and cis-regulatory elements annotated by rVarbase, their information about protein bound and motif change of TF that derived from HaploReg provided further evidence in support of their regulatory function (http://archive.broadinstitute.org/mammals/haploreg/)^52^. Based on the HaploReg database, disruption of TF binding motifs would be a major criterion for inclusion if SNPs were predicted to not bind the TF protein. SNPs that could disrupt more binding TF motifs were preferentially selected; if the number of binding TF motifs was identical, SNPs with greater change of motif LOD (log-odds) scores were selected. 3. The eQTL effects of SNPs on the brain regions derived from the Braineac and GTEx databases (http://www.braineac.org/; http://www.gtexportal.org/) were additive evidence to support their potential transcriptional regulatory function^52, 53^.

### SNP genotyping

Genomic DNA was extracted from the blood using a Qiagen QIAamp DNA Mini Kit (Qiagen, Hilden, Germany). The Agena Bioscience platform (http://www.agenabio.com), which is based on the matrix-assisted laser desorption/ionization time-of-flight (MALDI-TOF) primer extension assay, was used to genotype three SNPs (rs4836317, rs9327438 and rs6866678)^54^. We used the iPlex genotyping assay, which showed increased plexing efficiency and flexibility for the MassARRAY system via single base primer extension with mass-modified terminators^55^. All primers were designed according to the sequence of the forward strand from the dbSNP database (http://www.ncbi.nlm.nih.gov/SNP/). The remaining two SNPs (rs4836316 and rs2194079) were genotyped by direct Sanger DNA sequencing. The previous literatures reported that the genotyping accuracy of Agena Bioscience platform was up to 97.5%^56^. The raw accuracy of Sanger sequencing is 99.99%^57^. The primers that were required by the iPlex genotyping assay and Sanger sequencing are listed in Supplementary Table S2. The polymerase chain reaction (PCR) amplification in Sanger sequencing was performed in a 15 μL volume that contained 10 mM Tris-HCl (pH 8.3), 50 mM KCl, 1.5 mM MgCl_2_, 200 mM of each dNTPs, 0.3 mM of each primer, 1 U of Taq DNA polymerase, and 40 ng of the genomic DNA. PCR amplification was conducted with an initial denaturation phase at 94 °C for 5 min, followed by 38 cycles at 94 °C for 30 sec, with annealing at 59 °C for 30 sec, and extension at 72 °C for 40 sec, followed by a final extension phase at 72 °C for 7 min. DNA sequencing was performed after cleaning the PCR product using a BigDye Terminator Cycle Sequencing Ready Reaction Kit with Ampli Taq DNA polymerase (PE Biosystem). The inner primers were used for cycle sequencing, and the fragments were separated by electrophoresis on an ABI PRISM 377-96 DNA Sequencer (Applied Biosystem, Foster city, USA). In addition, in 10% of the samples that were randomly selected, Sanger sequencing was used to confirm the genotyping results derived from the Agena Bioscience platform.

### Statistical analyses

Deviation from the HWE for genotype frequency distributions was analyzed using the Chi-square goodness-of-fit test. Mendelian errors were detected by the family-based association test (FBAT) program version 2.0.3 (http://www.biostat.harvard.edu/fbat/default.html)^58^. Genotypes of families with Mendelian errors were reset to zero. Haploview version 4.2 (http://www.broad.mit.edu/mpg/haploview/) was used to calculate the values of pairwise *D*’ and *r* ^*2*^ for SNP pairs and construct haplotypes. SNP pairs were considered to be in strong linkage disequilibrium (LD) if *r* ^*2*^ > 0.80. We performed association tests using the FBAT program. The FBAT program implemented a generalized score statistic to perform a variety of transmission disequilibrium tests (TDT), including haplotype analysis. Additive and dominant inheritance models were examined in single marker association tests. The Bonferroni correction was used to reduce the rate of type I errors. The significance level was set to an alpha value of *p* < 0.01 (α/n = 0.05/5 = 0.01). Moreover, the global haplotype tests of association were performed under “multiallelic” mode in haplotype based association tests (HBAT). Meanwhile, the individual haplotype tests were conducted under “biallelic” mode in HBAT. A permutation test (n = 10,000) was used for multiple testing correction in HBAT, which is capable of recalculating the test statistic and reconstructing the distribution of the population. The empirical *p* value calculated by this test determines the significance level of results in the population. All *p* values calculated by the FBAT program were two-sided.

### Dual-luciferase reporter assay

The dual-luciferase reporter assay was conducted to validate the potential regulatory effects of the risk haplotype on transcriptional activity. First, a 1.5 Kb fragment that included rs4836316, rs2194079 and rs4836317 was generated by PCR. The forward and reverse primers were 5′***GAGCTC***TCACGCAAGGACCCACATTAT 3′ and 5′***CTCGAG***CCCACTTCATGCTTCTGGTATA 3′, respectively.

We used the template DNA from individuals containing the haplotype T-A-G (rs4836316-rs2194079-rs4836317). The resulting fragments were cloned into the *SacI* and *XhoI* sites of the pGL3-basic vector (Promega, Madison, WI, USA). Multiple site-directed mutagenesis was then used to obtain the construct containing the haplotype G-G-T (rs4836316-rs2194079-rs4836317). All DNA constructs were verified by sequencing. The pGL3-basic plasmid for background luminescence derived from the empty vector served as the control. The pGL3-promoter plasmid containing the SV40 promoter was used as a positive control. Transfection was performed using Lipofactamine 2000 (Life Technologies, Carlsbad, CA, USA) in triplicate for each construct. The 293T and C6 cells (at a density of 2 × 10^5^ each) were seeded into 12-well cell culture plates and co-transfected with pRL-TK (Promega, Madison, WI, USA). A pRL *Renilla* Luciferase Control Reporter Vector served as a normalizing control. After 24 h, cells were lysed with a passive lysis buffer and the luciferase activity was analyzed using the Dual-Luciferase Assay System (Promega, Madison, USA) on a luminometer (Turner Biosystems, Sunnyvale, USA). Reporter activity was measured as the ratio of firefly-to-*Renilla* luciferase activity. Cell-lines used in this assay were derived from the Cell Resource Center of Peking Union Medical College (PUMC). They were also confirmed not to be contaminated by mycoplasma. The value of fold induction over promoter, less the vector, in each group of different cells was compared by an independent t-test. Analyses above were conducted in SPSS v.20.0 statistical analysis software program. All *p* values calculated by these statistic tests were two-sided.

### Electrophoretic mobility shift assay

To investigate the role of the three putative functional SNPs (rs4836316, rs2194079 and rs4836317) in protein-binding, an electrophoretic mobility shift assay (EMSA) was performed using 30 bp probes encompassing the common variants of each of these 3 SNPs. The sense strands of the double-stranded probes included 5′ biotin-labeled oligonucleotides, which were annealed with the antisense strand to form the probes. The C6 cell nuclear extracts were incubated with the probes at room temperature for 20 min. The Light Shift Chemiluminescent EMSA kit (Pierce, Holmdel, USA) was used. The reaction mixture was separated by 8% polyacrylamide gel electrophoresis (PAGE), and the products were detected by Stabilized Streptavidin-Horseradish Peroxidase Conjugate (Pierce, Holmdel, USA). Unlabeled probes at a 100-fold molar excess were added to the reaction for competition.

### In silico analyses of *MEGF10* expression in human brain and the peripheral blood of autistic individuals

Three online databases that included BRAINEAC and GTEx were utilized to determine the expression pattern of *MEGF10* in humans. The GTEx databases revealed the expression of *MEGF10* in 53 human tissues or cells (including 13 brain regions)^53^. The Human Brain Transcriptome (HBT) databases (http://hbatlas.org/pages/hbtd) provided the figure related to dynamic *MEGF10* expression during development and adulthood in the cerebellar cortex, mediodorsal nucleus of the thalamus, striatum, amygdala, hippocampus and 11 areas of the neocortex^59^.

Meanwhile, a Gene Expression Omnibus (GEO) dataset from Alter *et al*. (https://www.ncbi.nlm.nih.gov/geoprofiles) provided the normalized expression value of *MEGF10* that was counted by the affymetrix array in the peripheral blood of 69 autistic individuals and 77 healthy controls^47, 60^. This study was designed to compare gene expression profiles in peripheral blood lymphocytes of children with autism and controls. Quantile normalization adjusted all data so that they had similar distribution patterns. The normalized expression values between the two groups were compared by independent t-test. Analyses above were conducted with SPSS v.20.0 software. All *p* values calculated by these statistical tests were two-sided.

## Electronic supplementary material

Supplementary information
