# Supplementary material for: Genetic variants in the transcription regulatory region of MEGF10 are associated with autism in Chinese Han population
Source: Sci Rep. 2017 May 23;7:2292. doi: 10.1038/s41598-017-02348-1 (PMC5442155; doi:10.1038/s41598-017-02348-1)
Supplement: Supplementary file 1 — Supplementary information [file 41598_2017_2348_MOESM1_ESM.pdf]

Genetic variants in the transcription regulatory region of *MEGF10* are associated with autism in Chinese Han population

Zhiliu Wu[1,2], Jian Qin[3], Yang You[1,2], Yuanlin Ma[4,5], Meixiang Jia[1,2], Linyan Wang[1,2] Tianlan Lu[1,2], Weihua Yue[1,2], Yanyan Ruan[1,2], Dai Zhang[1,2,4,5], Jun Li[1,2] and Lifang Wang[1,2].

[1]Institute of Mental Health, The Sixth Hospital, Peking University, Beijing, P. R. China.

[2] Key Laboratory of Mental Health, Ministry of Health & National Clinical Research Center for Mental Disorders (Peking University), Beijing, P. R. China.

[3]Central Laboratory, Renmin Hospital, Wuhan University, Wuhan, Hubei, P. R. China

[4]Peking-Tsinghua Center for Life Sciences, Peking University, Beijing, P. R. China.

[5]PKU-IDG/McGovern Institute for Brain Research, Peking University, Beijing, P. R. China.

Supplementary information index

Supplementary Figure S1. The haplotype block that covered the transcription start site region of *MEGF10*

Supplementary Figure S2. *MEGF10* expression in 10 human brain regions from the BRAINEAC database

Supplementary Figure S3. expression Quantitative trait loci (eQTL) data of 5 selected SNPs in 10 human brain regions from the BRAINEAC database

Supplementary Figure S4. *MEGF10* expression in 53 human tissues or cells from GTEx database

Supplementary Figure S5. Dynamic expression of *MEGF10* in the cerebellar cortex, mediodorsal nucleus of the thalamus, striatum, amygdala, hippocampus and 11 areas of neocortex

Supplementary Figure S6. *MEGF10* expression in the peripheral blood from autism individuals and healthy controls in Gene Expression Omnibus (GEO) pofile database

Supplementary Figure S7. A diagram of the position of 5 selected SNPs in *MEGF10*

Supplementary Table S1. The characteristic of SNPs in the haplotype that cover transcription start site (TSS) region of *MEGF10*

Supplementary Table S2. Primers of 5 selected SNPs in *MEGF10*

Supplementary Table S3. Information of the selected 5 SNPs in *MEGF10* and genotype frequencies in 410 autism trios of Han Chinese descendant

Supplementary Table S4. Association analyses of 5 SNPs in *MEGF10* in 410 trios using FBAT in a dominant model

Supplementary Table S5. Results of the multi-tissue eQTL comparison for five selected SNPs in brain regions with strong eQTL effects in the genotype-tissue expression (GTEx) database

Supplementary Table S6. Single nucleotide polymorphisms (SNPs) in *MEGF10* which are nominally associated with autism in CEU population of Psychiatric Genomic Consortium (PGC) database

Supplementary Figure S1. The haplotype block that covered the transcription start site region of *MEGF10*

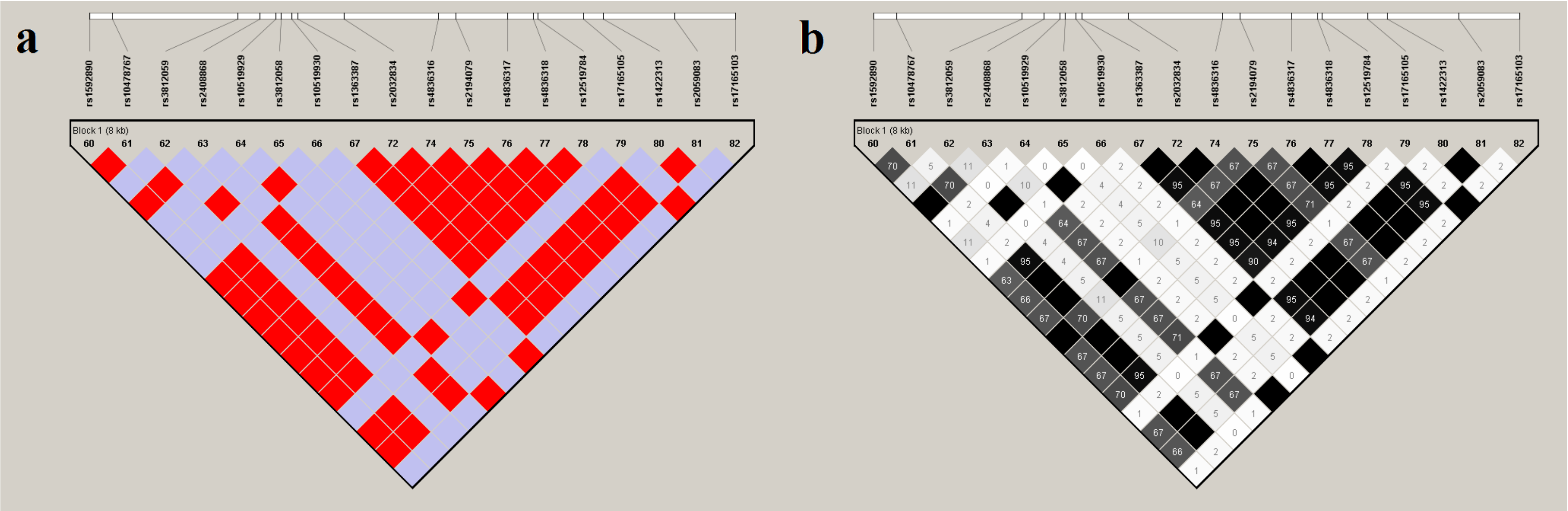

(a) Markers with linkage disequilibrium (LD) (D' < 1 and LOD > 2) are shown in red through pink (color intensity decreases with decreasing D' value). D' value shown in each square represents pairwise LD relationship between the two polymorphisms. D prime values of 1.0 are never shown (the box is empty). The LD plot was generated using the Haploview program.

(b) The value of r^2 is shown in each square. Black square indicated r^2 = 1(i.e. perfect LD between a pair of SNPs). Markers with LD are shown in black to grey (color intensity decreases with decreasing r^2 value).

Supplementary Figure S2. *MEGF10* expression in 10 human brain regions from the BRAINEAC database

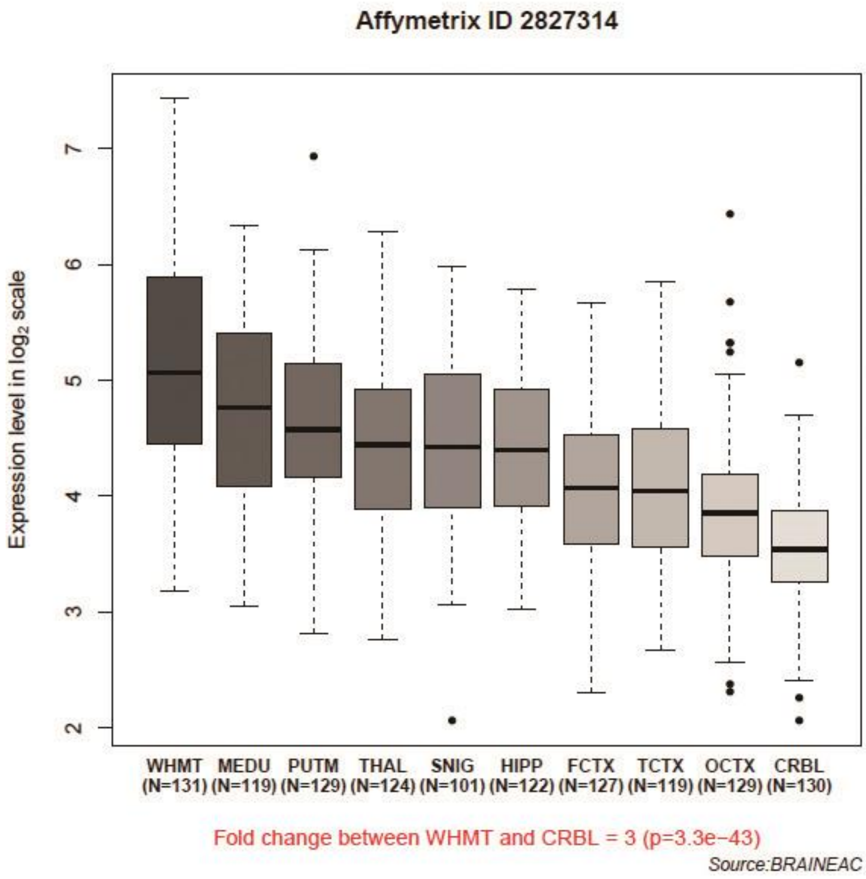

Abbreviations: WHMT: white matter; MEDU: medulla; PUTM: putamen; THAL: thalamus; SNIG: substantia nigra; HIPP: hippocampus; FCTX: frontal cortex; TCTX: temporal cortex; OCTX: occipital cortex; CRBL: cerebellum

Supplementary Figure S3. expression Quantitative trait loci (eQTL) data of 5 selected SNPs in 10 human brain regions from the BRAINEAC database

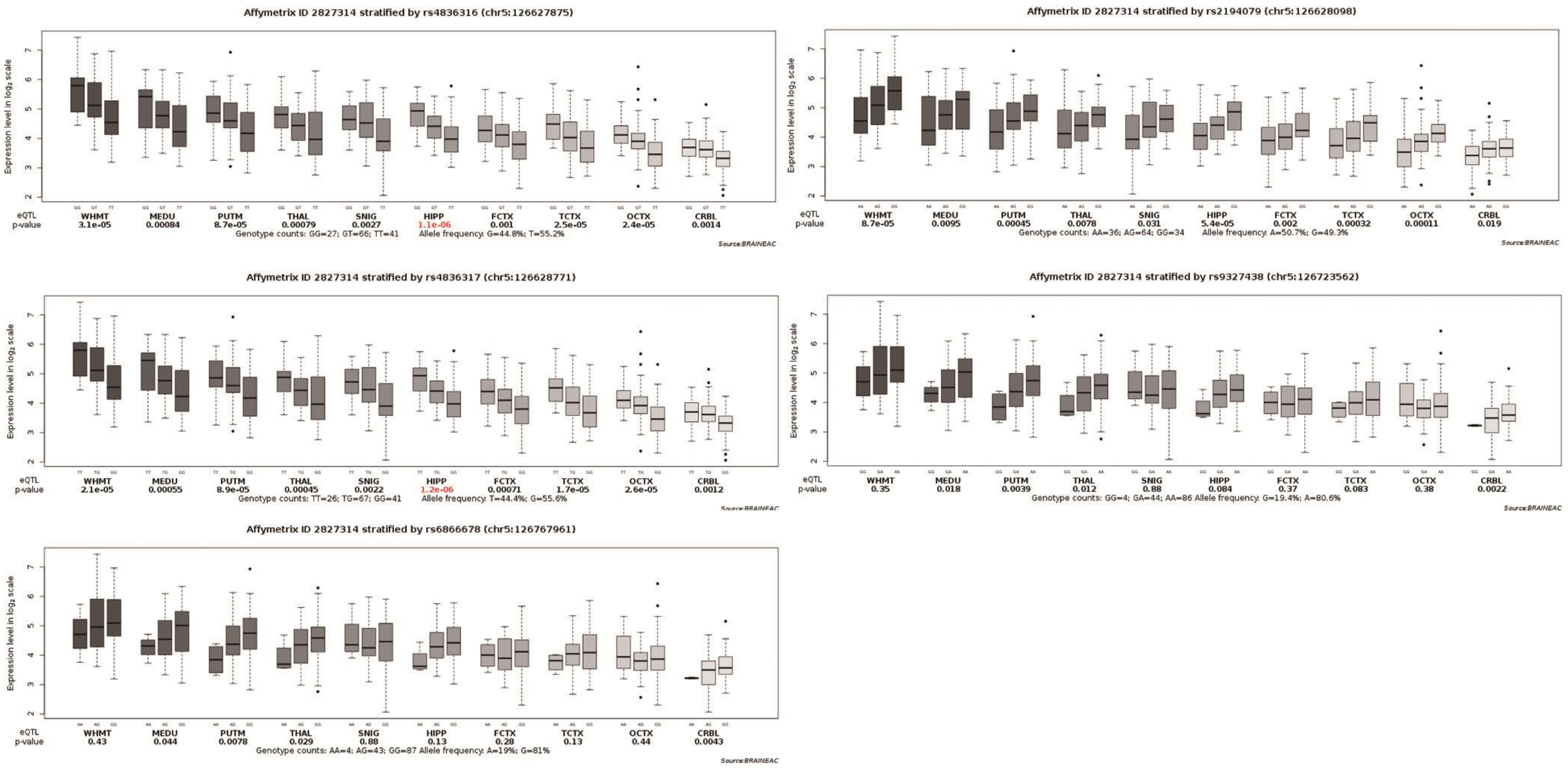

Abbreviations: WHMT: white matter; MEDU: medulla; PUTM: putamen; THAL: thalamus; SNIG: substantia nigra; HIPP: hippocampus; FCTX: frontal cortex; TCTX: temporal cortex; OCTX: occipital cortex; CRBL: cerebellum

Note: *P* value noted with Red color means that this significance persists after using FDR < 0.01 through the Benjamini-Hochberg procedure. The *P* value below 0.05 without red color means that these SNPs were nominally associated with expression of this gene in brain regions.

Supplementary Figure S4. *MEGF10* expression in 53 human tissues or cells from GTEx database

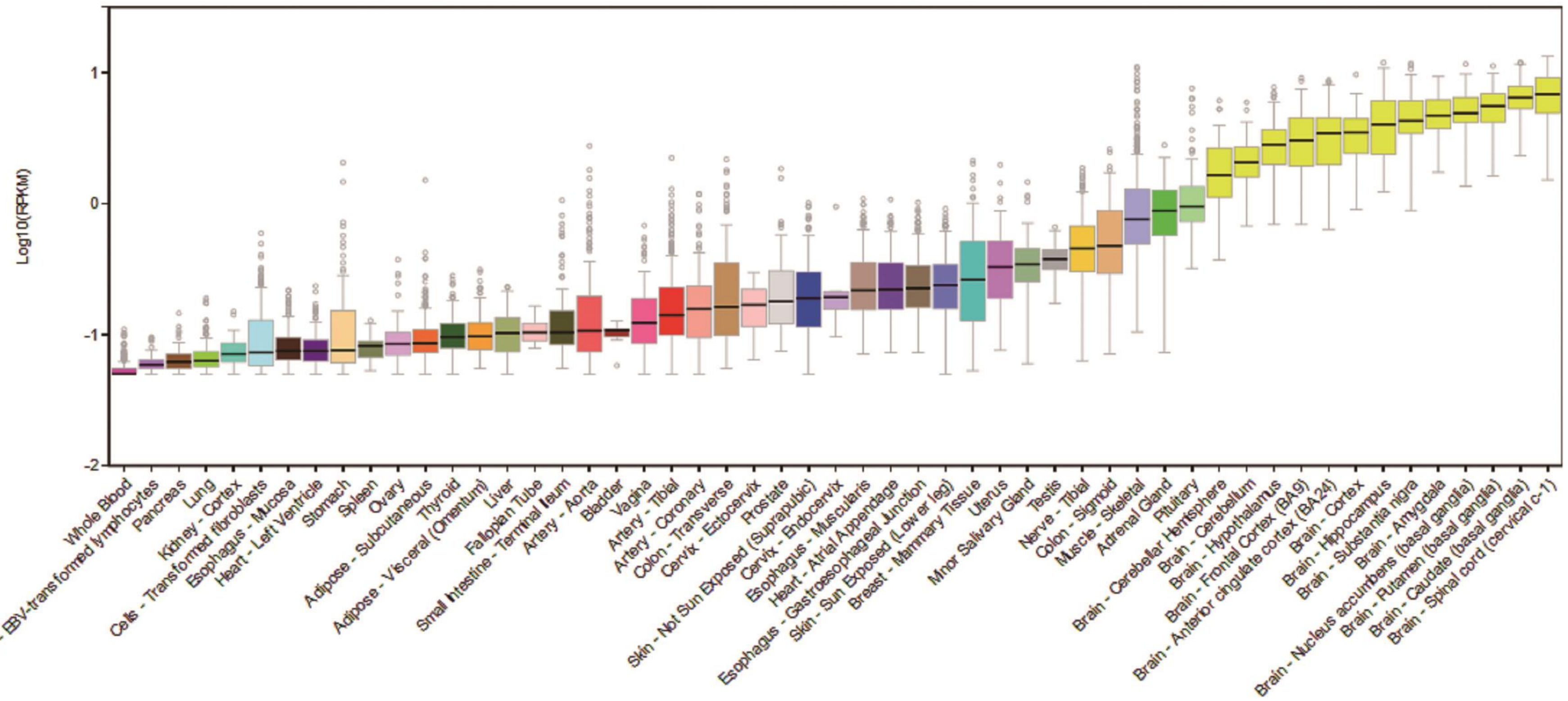

Abbreviations: RPKM: Reads Per Kilo bases per Million reads

Supplementary Figure S5. Dynamic expression of *MEGF10* in the cerebellar cortex, mediodorsal nucleus of the thalamus, striatum, amygdala, hippocampus and 11 areas of neocortex

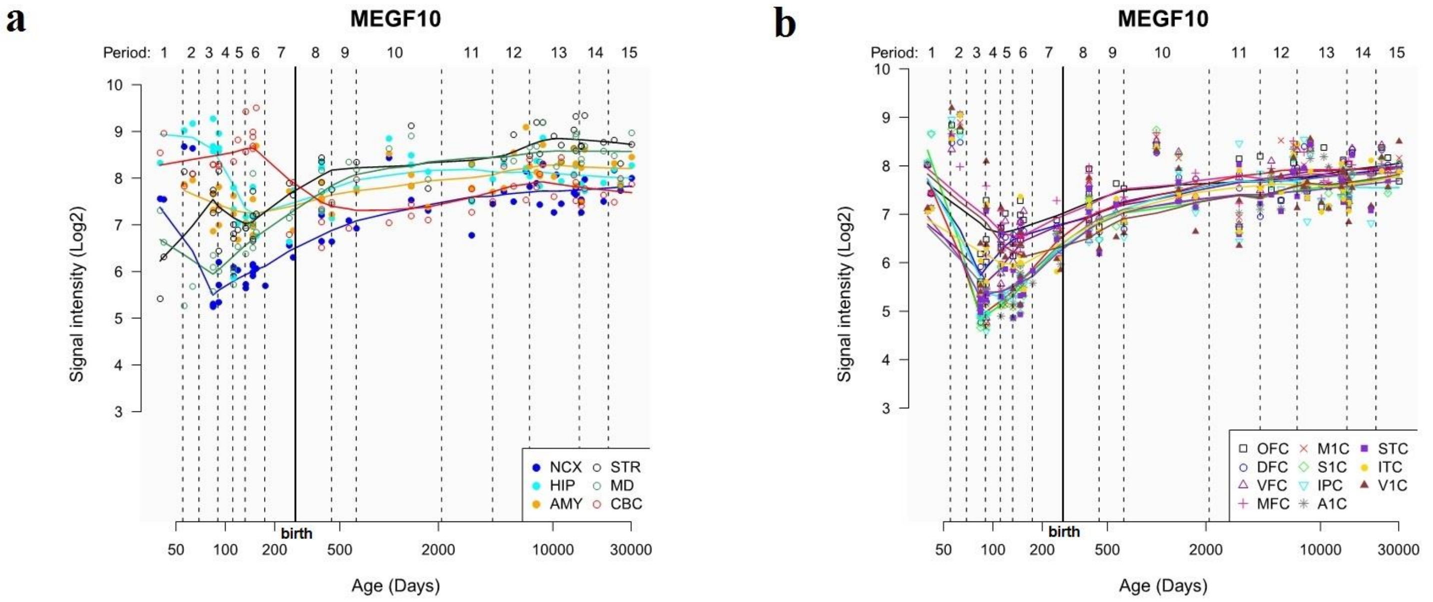

(**a**) Dynamic expression of *MEGF10* in 6 brain regions

Abbreviation: NCX: neocortex; STR: striatum; HIP: hippocampus; MD: mediodorsal nucleus; AMY: amygdala; CBC: cerebellar cortex

Note: solid line indicates the birth.

(**b**) Dynamic expression of *MEGF10* in 11 area of neocortex

Abbreviations: OFC: the orbital cortices; M1C: the primary motor cortices; STC: the posterior superior cortices; DFC: the dorsolateral cortices; S1C: the primary somatosensory cortices; ITC: the anterior inferior cortices; VFC: the ventrolateral cortices; IPC: the posterior inferior cortices; V1C: the primary visual cortex; MFC: the media cortices; A1C: the primary auditory cortices

Note: solid line indicates the birth.

Supplementary Figure S6. *MEGF10* expression in the peripheral blood from autism individuals and healthy controls in Gene Expression Omnibus (GEO) pofile database

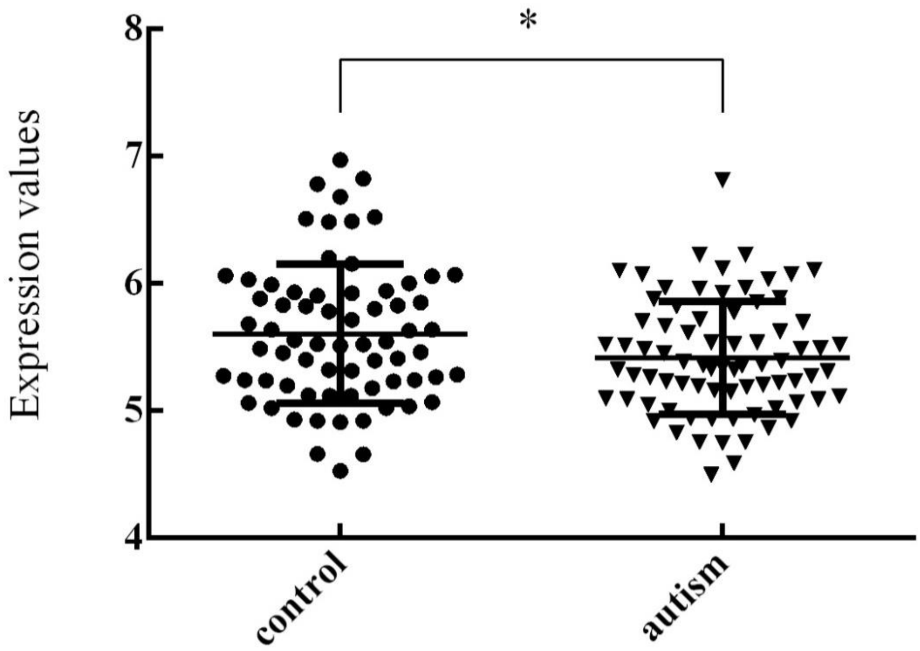

Note: Error bars represent mean ± standard deviation (SD); *: $P < 0.05$; Organism: Homo sapiens; Reporter: GPL570, 236517_at (ID_REF), GDS4431, 84466 (Gene ID), AI968440; DataSet type: Expression profiling by array, count, 146 samples; ID: 92265967. Expression values have been normalized by Quantile normalization.

Supplementary Figure S7. A diagram of the position of 5 selected SNPs in *MEGF10*

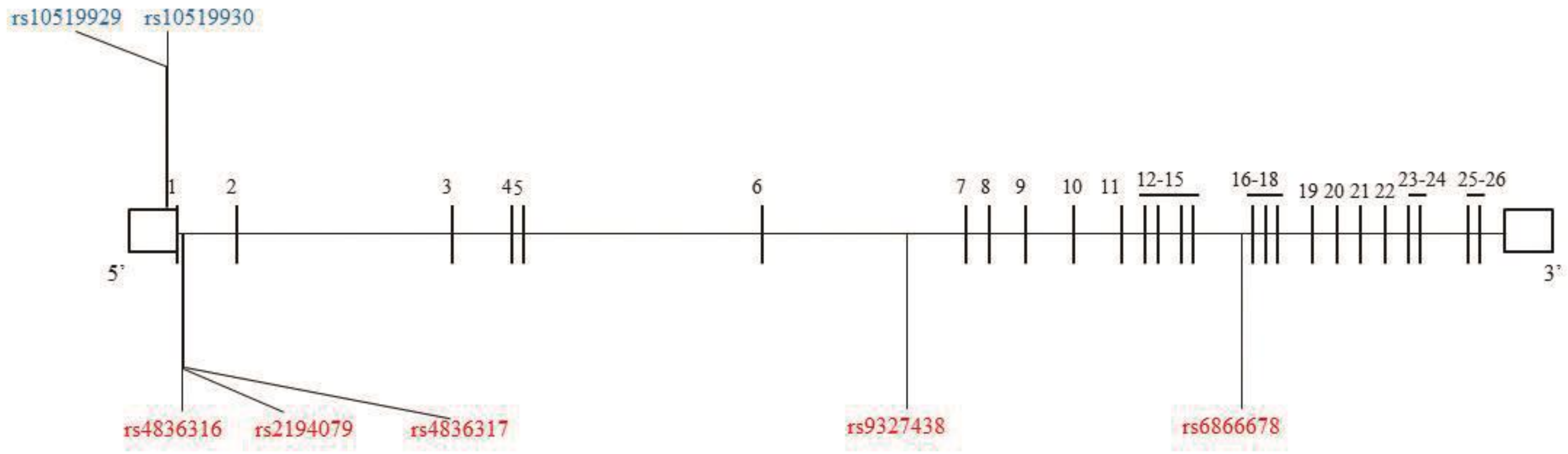

Note: Exons noted with number are in black; five selected SNPs are noted in red color; two SNPs in the transcription start site which are nominally associated with autism in the PGC samples are noted in blue color.

Supplementary Table S1. The characteristic of SNPs in the haplotype that cover transcription start site (TSS) region of *MEGF10*

SNP ID	Distance [1]	chromatin state	regulatory elements	proteins bound	motifs changed	eQTL signal
rs1592890	-3126 bp	8_ZNF/Rpts	n/a	n/a	Foxj2,Hoxa10,Hoxa9,Pou5f1, Sox	nominal
rs10478767	-2820 bp	8_ZNF/Rpts	n/a	n/a	PU.1,Pax-8	significant in HIPP
rs3812059	-1197 bp	7_Enh;8_ZNF/Rpts;	TF binding region	n/a	E2F,Nrf1,Pbx3	n/a
rs2408868	-908 bp	1_TssA;5_TxWk;7_Enh	TF binding region	n/a	Crx,Pax-4	nominal
rs10519929	-698 bp	1_TssA;5_TxWk;7_Enh; 12_EnhBiv	TF binding region	CTCF,RAD21, SMC3,NFKB	n/a	n/a
rs3812058	-629 bp	1_TssA;2_TssAFlnk;7_Enh; 11_BivFlnk;10_TssBiv;12_EnhBiv	TF binding region	CTCF,RAD21, SMC3,NFKB	CHD2, Hlx1, ZBTB33, Zfx	n/a
rs10519930	-492 bp	1_TssA;2_TssAFlnk;7_Enh; 11_BivFlnk;10_TssBiv;12_EnhBiv	TF binding region	NFKB,CTCF	n/a	n/a
rs1363387	-412 bp	1_TssA;2_TssAFlnk;7_Enh; 11_BivFlnk;10_TssBiv;12_EnhBiv	TF binding region	n/a	SIX5,VDR	significant in HIPP
rs2032834	+189 bp	1_TssA;2_TssAFlnk;7_Enh; 11_BivFlnk;10_TssBiv;12_EnhBiv	n/a	POL2,TAF1	n/a	significant in HIPP
rs4836316	+1419 bp	1_TssA;2_TssAFlnk;5_TxWk; 7_Enh;10_TssBiv;11_BivFlnk	chromatin interactive region	n/a	HNF4,Mrg1::Hoxa9,RAR,RX RA, TCF4,VDR	significant in HIPP
rs2194079	+1642 bp	1_TssA;2_TssAFlnk;5_TxWk; 7_Enh;10_TssBiv	chromatin interactive region	n/a	Gsc,Msx2	nominal
rs4836317	+2315 bp	1_TssA;2_TssAFlnk;5_TxWk; 7_Enh	n/a	n/a	CEBPG,Maf,RFX5,RXRA	significant in HIPP
rs4836318	+2656 bp	1_TssA;2_TssAFlnk;5_TxWk; 7_Enh	n/a	n/a	Foxd1,Foxk1,HNF1,Maf,Pax-4, RXR::LXR,Sox	significant in HIPP

rs12519784	+2714 bp	1_TssA;2_TssAFlnk;5_TxWk; 7_Enh	n/a	POU5F1,USF2	Foxj1,TATA	nominal
rs17165105	+3305 bp	1_TssA;2_TssAFlnk;5_TxWk; 7_Enh	n/a	n/a	ERalpha-a,FXR,GATA,HDAC 2,NR4A,Nanog,RORalpha1	n/a
rs1422313	+3560 bp	2_TssAFlnk;5_TxWk;7_Enh	n/a	n/a	Nkx2,Smad4	significant in HIPP
rs2059083	+4492 bp	5_TxWk;7_Enh	n/a	n/a	Dlx3	significant in HIPP
rs17165103	+5280 bp	5_TxWk;7_Enh	n/a	n/a	HNF1,Irf,Nanog,Sox,p300	n/a

Abbreviations: n/a: not applicable; HIPP:hippocampus; The complete term description of chromatin states, protein bounds and motifs changed has been showed in the following website: http://rv.psych.ac.cn/; http://archive.broadinstitute.org/mammals/haploreg/haploreg.php

Note: [1] The distance from the TSS of *MEGF10*; "-": upstream of TSS of *MEGF10*; "+": downstream of TSS of *MEGF10*; the information of chromatin states and regulatory elements were from the rVarbase database; the information of proteins bound and motifs changed were from the HaploReg database; The eQTL data were from the Braineac database.

Supplementary Table S2. Primers of 5 selected SNPs in *MEGF10*

Genotyping methods	Marker	Information of PCR primers	Information of extension primers
Sanger sequencing	rs4836316	U: 5’ GTGATAACCATGCTGTGCCAAG 3’	n/a
	rs2194079	L: 5’ ACAGTCTTCAAAGTCCATCCAAAT 3’	n/a
Sequenom platform	rs4836317	U: 5’ ACGTTGGATGTTCCCTATGACCTCAAGCAG 3’	5’ CCCAAGTTTATACATAATAAAACTGATG 3’
		L: 5’ ACGTTGGATGTCCCCACCTCTCAACACAAG 3’	
	rs9327438	U: 5’ ACGTTGGATGTATCTTAGAAAGCCTCCCGC 3’	5’ GCTCCACTCTCTGTTTC 3’
		L: 5’ ACGTTGGATGTTCTTCTCTCCTAACCTGCC 3’	
	rs6866678	U: 5’ ACGTTGGATGTTGCTATTCCAGTGTTCACC 3’	5’ GGGCCAAACTATAATGTGAATGTTG 3’
		L: 5’ ACGTTGGATGCTTAACATGGAAGCAGTCCC 3’	

Abbreviations: U: upper primer; L: Lower primer; n/a: not applicable.

Supplementary Table S3. Information of the selected 5 SNPs in *MEGF10* and genotype frequencies in 410 autism trios of Han Chinese descendant

Marker	Chromosome	Genotype frequencies in children			p_{HWE} [1]	Genotype frequencies in parents			p_{HWE} [2]
rs4836316	5:127292183	TT	TG	GG	0.16	TT	TG	GG	0.80
		213	172	24		397	343	78	
rs2194079	5:127292406	AA	AG	GG	0.52	AA	AG	GG	0.33
		147	201	60		265	413	140	
rs4836317	5:127293079	GG	GT	TT	0.14	GG	GT	TT	0.54
		207	171	24		394	340	81	
rs9327438	5:127387870	GG	GA	AA	0.85	GG	GA	AA	0.51
		157	187	58		287	399	126	
rs6866678	5:127432269	AA	AG	GG	0.23	AA	AG	GG	0.17
		134	187	83		227	426	165	

Note: [1] Hardy-Weinberg equilibrium *p* value for genotype distributions in children affected with autism; [2] Hardy-Weinberg equilibrium *p* value for genotype distributions in parents.

Supplementary Table S4. Association analyses of 5 SNPs in *MEGF10* in 410 trios using FBAT in a dominant model

Marker	Chromosome	Allele	Allele frequency	Fam	S-E (S)	Var (S)	Z	*P*
rs4836316	5:127292183	T	0.707	103	12.75	21.31	2.762	**0.0057**
		G	0.293	237	-17.25	54.81	-2.330	0.020
rs2194079	5:127292406	A	0.585	177	10.50	38.00	1.703	0.089
		G	0.415	232	-16.00	51.75	-2.224	0.026
rs4836317	5:127293079	G	0.707	100	12.50	20.63	2.752	**0.0059**
		T	0.293	236	-15.50	54.63	-2.097	0.036
rs9327438	5:127387870	G	0.609	147	9.25	31.19	1.656	0.098
		A	0.391	242	-12.25	54.94	-1.653	0.098
rs6866678	5:127432269	A	0.547	189	4.00	40.38	0.630	0.53
		G	0.453	230	-16.50	50.63	-2.319	0.020

Abbreviations: Afreq, allele frequency; Fam, number of informative families; S, test statistics for the observed number of transmitted alleles; E(S), expected value of S under the null hypothesis (i.e., no linkage and no association).

Note: *P* value in bold font indicates persistent statistical significance after the Bonferroni correction.

Supplementary Table S5. Results of the multi-tissue eQTL comparison for five selected SNPs in brain regions with strong eQTL effects in the genotype-tissue expression (GTEx) database

SNP ID	Tissue	Samples	Beta	*p*-value	post-prob
rs4836316	Cerebellum	103	0.401	5.9e-4	0.999
	Frontal cortex	92	0.365	1.2e-3	1.000
	Cerebellar hemisphere	89	0.341	0.006	1.000
	Cortex	96	0.299	0.01	0.987
	Caudate	100	0.257	0.01	0.981
	Putamen	82	0.210	0.05	0.971
rs2194079	Frontal cortex	92	0.434	1.7e-4	0.992
	Cerebellum	103	0.404	7.3e-4	0.998
	Cortex	96	0.390	2.1e-3	0.999
	Cerebellar hemisphere	89	0.376	2.2e-3	0.988
	Caudate	100	0.269	0.009	0.988
	Putamen	82	0.234	0.03	0.947
rs4836317	Cerebellum	103	0.395	6.5e-4	0.991
	Frontal cortex	92	0.353	1.4e-3	0.999
	Cerebellar hemisphere	89	0.341	0.006	0.994
	Cortex	96	0.285	0.02	0.986
	Caudate	100	0.281	0.006	0.994
	Putamen	82	0.205	0.05	0.960
rs9327438	Frontal cortex	92	0.399	0.007	0.982
	Anterior cingulate cortex	72	0.399	0.04	0.951
	Cerebellum	103	0.374	0.03	0.961
	Putamen	82	0.333	0.01	0.980
	Hypothalamus	81	0.333	0.03	0.957
	Nucleus accumbens	93	0.293	0.03	0.970
rs6866678	Anterior cingulate cortex	72	0.416	0.03	0.958
	Frontal cortex	92	0.354	0.02	0.981
	Putamen	82	0.315	0.02	0.987
	Nucleus accumbens	93	0.271	0.04	0.972

Abbreviations: post-prob: posterior probability.

Note: If post-prob is greater than 0.9, it means that the tissue is predicted to HAVE an eQTL effect. *p*-value: from a t-test that compared observed beta from single tissue eQTL analysis to a null beta of 0. A total of 53 human tissues including 10 brain regions were compared with cross-tissue meta-analysis. Here is listed the brain regions with post-prob > 0.9

Supplementary Table S6. Single nucleotide polymorphisms (SNPs) in *MEGF10* which are nominally associated with autism in CEU population of Psychiatric Genomic Consortium (PGC) database

SNP ID	Chromosome Position	Ref	Alt	OR	SE	*P* value	CEU af
rs10519929	5:126653657	A	G	1.1520	0.0677	0.03683	0.9633028
rs10519930	5:126653863	T	C	1.1760	0.0693	0.01923	0.9675926
rs17165105	5:126657660	T	C	0.8685	0.0679	0.03778	0.0366972
rs17165082	5:126664681	T	C	0.8354	0.0796	0.02398	0.0277778
rs246889	5:126685331	A	T	0.9292	0.0294	0.01248	0.6284400
rs17673147	5:126701241	A	C	0.8229	0.0589	0.00093	0.0366972
rs17165041	5:126704453	A	G	1.2090	0.0579	0.00105	0.9633028
rs187514	5:126711315	T	C	1.0820	0.0296	0.00766	0.3577980
rs27388	5:126711708	A	G	0.9444	0.0291	0.04903	0.6018520
rs17165037	5:126721657	A	G	0.8808	0.0479	0.00803	0.0825688
rs17165035	5:126729089	A	G	0.8525	0.0431	0.00022	0.0917431
rs17604615	5:126738855	T	C	0.9032	0.0407	0.01240	0.1146790
rs1363388	5:126741505	A	G	1.1030	0.0366	0.00753	0.8348620
rs10519936	5:126747744	T	C	0.9034	0.0366	0.00553	0.1651380
rs6881894	5:126750758	A	G	1.1010	0.0364	0.00798	0.8348620
rs10519938	5:126751220	T	G	1.0960	0.0387	0.01748	0.8486240
rs12233927	5:126765343	T	C	1.1160	0.0369	0.00302	0.8348620
rs17165090	5:126768215	A	G	0.9076	0.0365	0.00796	0.1651380
rs3844184	5:126773129	A	T	0.9001	0.0367	0.00418	0.1651380
rs3851469	5:126774333	A	G	0.8949	0.0367	0.00247	0.1651380
rs11950427	5:126797045	T	C	1.0910	0.0395	0.02744	0.8486240
rs6859304	5:126800963	A	G	1.0770	0.0289	0.01057	0.6467890
rs12516897	5:126808111	A	G	1.0710	0.0288	0.01790	0.6481480
rs10519941	5:126808243	T	C	1.0720	0.0288	0.01598	0.6467890
rs10072587	5:126810224	A	T	1.0810	0.0289	0.00733	0.6467890
rs17164935	5:126819181	A	G	0.8864	0.0385	0.00175	0.1284400
rs11743165	5:126819748	A	G	0.8481	0.0793	0.03775	0.0137615
rs11743831	5:126819982	C	G	1.1800	0.0808	0.04058	0.9862385
rs17673685	5:126820248	A	G	1.098	0.0428	0.02832	0.8853210
rs3756721	5:126823058	A	G	0.9018	0.0362	0.00435	0.1651380

Abbreviations: Ref: reference; Alt: alteration; OR: odds ratio; SE: standard error; CEU af: allele frequency in Northern and Western European Ancestry in Utah
